# Role of Dendritic Cells in Mediating the Effect of Growth Differentiation Factor 15 on Nonalcoholic Fatty Liver Disease: Insights From Causal Inference and Single-Cell Profiling

**DOI:** 10.1155/mi/1153091

**Published:** 2025-11-24

**Authors:** Tao Yao, Peng Zheng, Zhe-Ning Wang, Luo-Xiang Fang, Yu-Huan Wu, Yuan-Nan Lin

**Affiliations:** ^1^Department of Cardiology, The Second Affiliated Hospital and Yuying Children's Hospital of Wenzhou Medical University, Wenzhou 325000, Zhejiang, China; ^2^Department of Gastroenterology, Zhejiang Chinese Medical University The Fourth School of Clinical Medicine, Hangzhou 310053, Zhejiang, China

**Keywords:** growth differentiation factor 15, immune cells, mediation analysis, Mendelian randomization, nonalcoholic fatty liver disease

## Abstract

**Background:**

Nonalcoholic fatty liver disease (NAFLD) represents one of the most prevalent chronic liver conditions worldwide. Previous studies have highlighted an association between circulating growth differentiation factor 15 (GDF-15) levels and NAFLD, as well as the involvement of immune cells in its pathogenesis and progression. However, the causal relationships remain unclear.

**Methods:**

We obtained summary-level data for circulating GDF-15 levels, NAFLD, and 731 immune cell phenotypes from large-scale genome-wide association studies (GWAS) and the FinnGen database. Mendelian randomization (MR) analysis was employed to explore the causal relationship between circulating GDF-15 levels and NAFLD. Additionally, a two-step MR approach was utilized to identify and assess the mediatory role of immune cells in this association. Finally, single-cell RNA sequencing analysis (scRNA-seq) was performed to validate the proportions of CD123^+^ dendritic cell (DC) subsets in NAFLD progression.

**Results:**

Two-sample MR analysis revealed that elevated levels of GDF-15 are associated with an increased risk of NAFLD (OR = 1.16; 95% confidence interval [CI] = 1.03–1.30; *p*=0.017), and replication analysis further confirmed the stability of these findings (OR = 1.10; 95% CI = 1.01–1.20; *p*=0.037). Mediation analysis identified that CD123 on plasmacytoid DCs (DCs), CD123 on CD62L+ plasmacytoid DCs, CD80 on plasmacytoid DCs, and CD80 on CD62L+ plasmacytoid DCs mediate the causal effect of GDF-15 on NAFLD. Sensitivity analyses and bidirectional MR further ensured the robustness of these findings. Single-cell analysis further validated these results.

**Conclusions:**

Our findings propose a causal relationship between GDF-15 and NAFLD mediated by DCs, offering novel insights for potential therapeutic and preventive strategies for NAFLD.

## 1. Introduction

Nonalcoholic fatty liver disease (NAFLD), a widespread chronic liver condition, ranges from simple steatosis to nonalcoholic steatohepatitis (NASH), liver fibrosis, cirrhosis, and, in some cases, hepatocellular carcinoma (HCC [1]). Beyond the liver, NAFLD is intricately linked to extrahepatic conditions such as cardiovascular disease (CVD), chronic renal failure, and certain malignancies, significantly amplifying its overall disease burden [2]. The global obesity epidemic has fueled a rapid increase in NAFLD prevalence, with an estimated 25.24% of adults affected worldwide [3]. Despite this significant health burden, no approved pharmacotherapies directly target NAFLD pathogenesis, underscoring an urgent need to elucidate its underlying mechanisms.

Growth differentiation factor 15 (GDF-15), a divergent member of the transforming growth factor-β superfamily, functions as a stress-responsive cytokine [4, 5]. It is connected to a range of biological processes, including tumorigenesis, inflammatory responses, and tissue injury [6–8]. The relationship between GDF-15 and NAFLD remains uncertain. Observations indicated that GDF-15 levels were elevated in the bloodstream of NAFLD patients [9]. Additionally, some evidence suggests that GDF-15 may act as a profibrotic factor in the progression of liver fibrosis [10]. However, these associations could be influenced by reverse causation, confounding factors, or selection bias; thus, whether GDF-15 plays a causal role in NAFLD development is unknown and warrants investigation.

NAFLD is traditionally viewed as a metabolic disorder; however, its progression is also closely linked to inflammation mediated by immune cells [11]. Evidence suggests that immune cells modulate liver disease progression by secreting inflammatory cytokines that exert either pro-inflammatory or anti-inflammatory effects, directly or indirectly [12]. Meanwhile, GDF-15 is an immunoregulatory factor that targets immune cells to inhibit excessive inflammatory responses, according to studies [13, 14]. Therefore, immune cells have the potential to act as mediators in the relationship between GDF-15 and NAFLD.

Mendelian randomization (MR) uses genetic variants as instrumental variables (IVs) to determine causal links between exposures and clinical outcomes, effectively mitigating potential confounding and reverse causation bias [15]. This study's objectives are to explore the causal relationship between circulating GDF-15 levels and NAFLD using MR and to assess the mediating function of immune cells in the GDF-15–NAFLD axis through mediation analysis. Defining the GDF-15–immune cell–NAFLD axis could deepen our understanding of NAFLD pathogenesis and provide robust evidence for developing feasible screening and prevention strategies for NAFLD.

## 2. Method

### 2.1. Study Design

**Figure 1:**
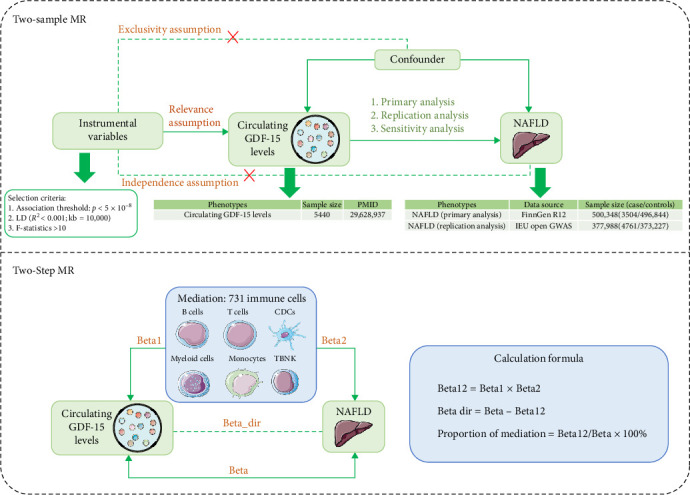
Study design overview. NAFLD, nonalcoholic fatty liver disease; GDF-15, growth differentiation factor 15.

This study employed genome-wide association studies (GWAS) and multiple MR analysis methods to examine the causal relationship between GDF-15 and NAFLD, along with potential immunological mediation. The study was conducted in two main stages. In the first stage, two-sample MR was applied to ascertain whether GDF-15 and NAFLD are causally related. To enhance the reliability of the results, two independent NAFLD datasets were utilized, with one serving as the primary analysis and the other for replication to validate the findings. In the second stage, potential mediators of this causal relationship were identified among 731 immune cell phenotypes, along with their mediating effects. Finally, single-cell RNA sequencing (scRNA-seq) data were analyzed to validate the changes in CD123^+^ dendritic cells (DCs) during NAFLD progression (Figure 1).

### 2.2. Data Sources

The genetic data for circulating GDF-15 levels were sourced from a GWAS that integrated data from four community-based cohorts, comprising 5440 individuals of European ancestry ([16] Supporting Information 1: Table S1). Serum or plasma concentrations of GDF-15 were quantified using validated immunoassays.

The GWAS data for NAFLD used in the primary analysis were obtained from the FinnGen consortium (r12 version), comprising 3504 NAFLD cases and 496,844 controls of European ancestry ([17] Supporting Information 1: Table S1). In the FinnGen database, NAFLD was defined as the fatty replacement of the hepatic parenchyma unrelated to alcohol use, classified under the ICD-10 code K76.0. For replication analysis, summary statistics for NAFLD GWAS were derived from a recently published dataset in the GWAS Catalog (GCST90054782), which included 4761 NAFLD cases and 373,227 controls of European descent ([18] Supporting Information 1: Table S1).

The data on 731 immune cell phenotypes were derived from a study conducted by Orru et al. [19], which included 3757 individuals of European Sardinian ancestry (Supporting Information 1: Table S1). These immune cell phenotypes were divided into seven groups, including monocytes, myeloid cells, T cells, regulatory T cells (Treg), B cells, conventional DCs (cDCs), T cells, and TBNK (T cells, B cells, and natural killer cells [NKT cells]). Four types of measurements were included in the dataset, as detailed in Supporting Information 1: Table S2. These phenotypes were assessed using standardized flow cytometry techniques, with peripheral blood samples stained and analyzed on BD FACSCanto II machines. These 731 immune cell phenotypes, which include more than 22 million genetic variations, have GWAS summary statistics that are publicly accessible in the GWAS Catalog (Accession codes GCST0001391–GCST0002121).

### 2.3. Instrumental Variable Selection

To guarantee the robustness of the results, a stringent IV selection process was implemented, as described previously [20]. First, SNPs associated with circulating GDF-15 levels and 731 immune cell phenotypes were identified using a genome-wide significance threshold of *p* < 5 × 10^−8^. Due to the small number of SNPs identified for immune cell phenotypes at this threshold, we relaxed the criterion to *p* < 5 × 10^−6^. Second, linkage disequilibrium (LD) was addressed to ensure independence among SNPs by applying a threshold of *r*^2^ < 0.001 and a distance of 10,000 kb. Third, after harmonizing the IVs with summary statistics from the outcome GWAS, SNPs associated with NAFLD were excluded, followed by the removal of palindromic SNPs to maintain consistency in the alignment of effect alleles and their effect sizes. Finally, weak instruments with F-statistics < 10 were excluded to reduce potential bias. The F-statistic was calculated as *F* = *β*^2^/SE^2^ [21].

### 2.4. Statistical Analysis

#### 2.4.1. MR Analysis

As the primary analysis, the random-effects inverse-variance weighted (IVW) method was employed, integrating each SNP's Wald ratio on the outcome to achieve a pooled causal estimate [22]. In order to obtain more robust estimates, we conducted supplementary MR analyses, including weighted median, MR-Egger, weighted mode, and simple mode. Weighted median estimates remain consistent even if up to 50% of genetic variants are invalid instruments [23]. MR-Egger, although having the lowest power, is capable of producing consistent estimates even when all the instruments are invalid, by accounting for pleiotropy [24]. The weighted mode method identifies clusters of SNPs with similar causal effect estimates and derives the causal effect from the largest and most coherent cluster, ensuring robustness against outliers and invalid instruments [25]. The simple mode method, similar to the weighted mode, clusters SNPs based on the similarity of their causal effect estimates but employs a straightforward unweighted averaging approach, making it easier to implement yet more susceptible to bias if invalid instruments dominate [26]. Results are deemed statistically significant and robust, warranting inclusion in further analyses, when the IVW method produces an estimate with a *p*-value below 0.05 and all methods demonstrate consistent effect directions.

To further enhance the robustness of our findings, we additionally incorporated three recently developed MR approaches: constrained maximum likelihood with model averaging (cML-MA), which combines constrained likelihood estimation with model averaging to provide unbiased causal estimates even in the presence of correlated and uncorrelated pleiotropy [27]; MR-Contamination Mixture (ConMix), which identifies groups of genetic variants with similar causal estimates and infers the causal effect from the valid subset, offering robustness when some IVs are invalid [28]; and MR-Robust Adjusted Profile Score (RAPS), which improves reliability by accounting for weak instruments, systematic pleiotropy, and outliers [29].

Moreover, given that body mass index (BMI) is one of the most important risk factors for NAFLD, we conducted multivariable MR (MVMR) analyses adjusting for BMI (data source: IEU GWAS ID ieu-b-4815, sample size = 51,892; Supporting Information 1: Table S1) to disentangle the direct effect of GDF-15 on NAFLD from potential confounding or mediation by BMI, thereby ensuring that the observed association was not merely attributable to the well-established effect of BMI.

#### 2.4.2. Sensitivity Analysis

We undertook various sensitivity analyses to test the durability of our results. Heterogeneity among the selected SNPs was evaluated using Cochran's Q test, with a *p* < 0.05 indicating significant heterogeneity [30]. The MR-Egger intercept test was conducted to detect directional pleiotropy, where a significant intercept (*p* < 0.05) suggests bias due to pleiotropic effects [24]. Horizontal pleiotropy was further evaluated using the MR-PRESSO global test, which identifies and corrects for outlier SNPs that could affect the results [31]. We performed leave-one-out analysis, removing each SNP one at a time and recalculating the IVW estimations in order to evaluate the impact of specific SNPs [32].

#### 2.4.3. Reverse Causality Analysis

We conducted reverse MR analysis for NAFLD and GDF-15, NAFLD and the identified immune cells, as well as the identified immune cells and GDF-15. The IV selection process is the same as before.

#### 2.4.4. Mediation Analysis

We employed a two-step MR approach to evaluate the mediating function of immune cell phenotypes in the causal relationship between GDF-15 and NAFLD. In the first step, two-sample MR was used to establish the causal association between GDF-15 and NAFLD and to quantify the effect size (Beta). In the second step, immune cell phenotypes causally associated with NAFLD (Beta2) were identified, followed by MR analysis between GDF-15 and the identified immune cell phenotypes (Beta1). Immune cell mediators were retained based on the consistency of the effect directions. We calculated the mediation effect (Beta1 × Beta2) and the proportion of the total effect attributable to the immune cell mediation ([Beta1 × Beta2]/Beta). Finally, the 95% confidence intervals (CI) and *p*-values for the mediation effect were then estimated using the delta approach [33]. A significant mediation effect was considered to exist if the *p*-value was less than 0.05.

### 2.5. Single-Cell Sequencing Analysis

To investigate the role of CD123^+^ DC subsets in NAFLD progression, we reanalyzed a public scRNA-seq dataset (GSE129516, *n* = 6), which included three biological replicates each from control (chow-fed) and NASH murine models [34]. Stringent quality control criteria were applied to ensure data reliability: cells with >5% mitochondrial gene content or expressing fewer than 200 or more than 10,000 genes were excluded. After filtering, 27,771 high-quality cells were retained for downstream analyses. The data were log-normalized, and the top 2000 highly variable genes were identified using the FindVariableFeatures function. Dimensionality reduction was performed by principal component analysis, and batch effects were corrected using the RunHarmony algorithm. Cells were clustered at a resolution of 0.4 and annotated based on canonical marker genes and lineage characteristics [35]. Subsequently, the proportions of Il3ra^+^ (murine CD123^+^) DCs were compared between groups to elucidate the potential role of CD123^+^ DCs in the pathogenesis and progression of NAFLD.

## 3. Results

### 3.1. Instrumental Variable Selection

After a stringent selection process for IVs, we identified 5 SNPs associated with circulating GDF-15 levels (*p* < 5 × 10^−8^), as presented in Supporting Information 1: Table S3. Additionally, 11,836 SNPs were selected as IVs for immune cell phenotypes (*p* < 5 × 10^−6^), as listed in Supporting Information 1: Table S4. The F-statistics for all IVs were greater than 10.

### 3.2. Causal Effect of Circulating GDF-15 Levels on NAFLD

**Figure 2:**
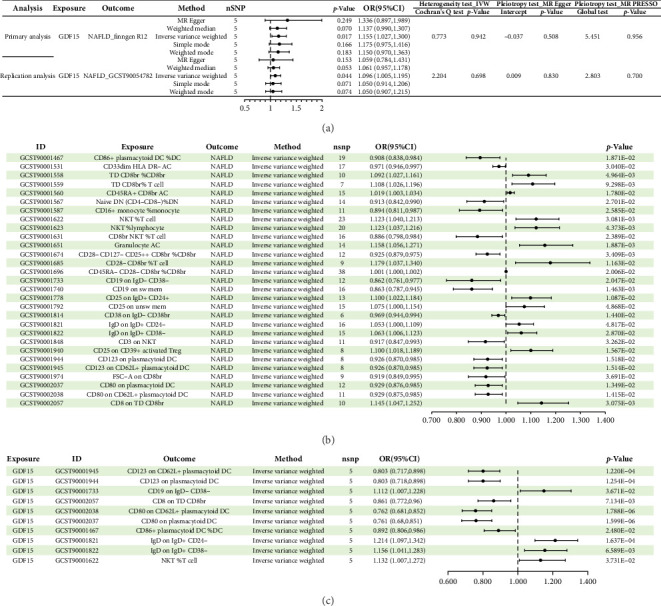
Forest plot. (A) MR results of causal associations between circulating GDF-15 levels and NAFLD. (B) Forest plot showing the causal associations between immune cell phenotypes and NAFLD. (C) Forest plot showing the causal associations between circulating GDF-15 levels and the identified immune cells. NAFLD, nonalcoholic fatty liver disease; GDF-15, growth differentiation factor 15; OR, odds ratio; CI, confidence interval.

The IVW approach demonstrated a causal correlation between elevated circulating GDF-15 levels and a higher risk of NAFLD (OR = 1.16; 95% CI = 1.03–1.30; *p*=0.017) in the primary analysis. Although the other four MR methods did not yield statistically significant results, the directions of the odds ratios were consistent with those observed in the IVW analysis. In addition, three complementary MR approaches—cML-MA (OR = 1.16; 95% CI = 1.03–1.30; *p*=0.018), MR-ConMix (OR = 1.17; 95% CI = 1.04–1.40; *p*=0.022), and MR-RAPS (OR = 1.16; 95% CI = 1.02–1.31; *p*=0.020)—all supported the positive association between circulating GDF-15 and NAFLD risk (Supporting Information 1: Table S5). MVMR analysis, adjusting for BMI, further confirmed this causal effect (OR = 1.16; 95% CI = 1.03–1.31; *p*=0.014), indicating the robustness of the findings. All sensitivity analyses showed no evidence of directional pleiotropy, heterogeneity, or outlier SNPs, and confirmed the robustness of the results. Similarly, replication analysis using an independent NAFLD GWAS corroborated these findings, suggesting a potential adverse effect of GDF-15 on the risk of NAFLD (OR = 1.10; 95% CI = 1.01–1.20; *p*=0.037). These results are summarized in Figure 2A and Supporting Information 2: Figures S1 and S2.

### 3.3. Mediator Screening of Immune Cells

#### 3.3.1. Causal Effect of Immune Cells on NAFLD

**Figure 3:**
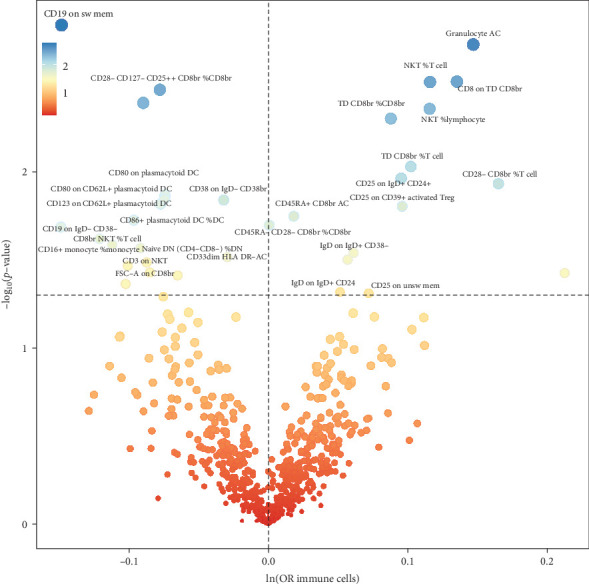
Volcano plot of immune cell associated with NAFLD based on two-sample MR analysis (IVW method).

**Figure 4:**
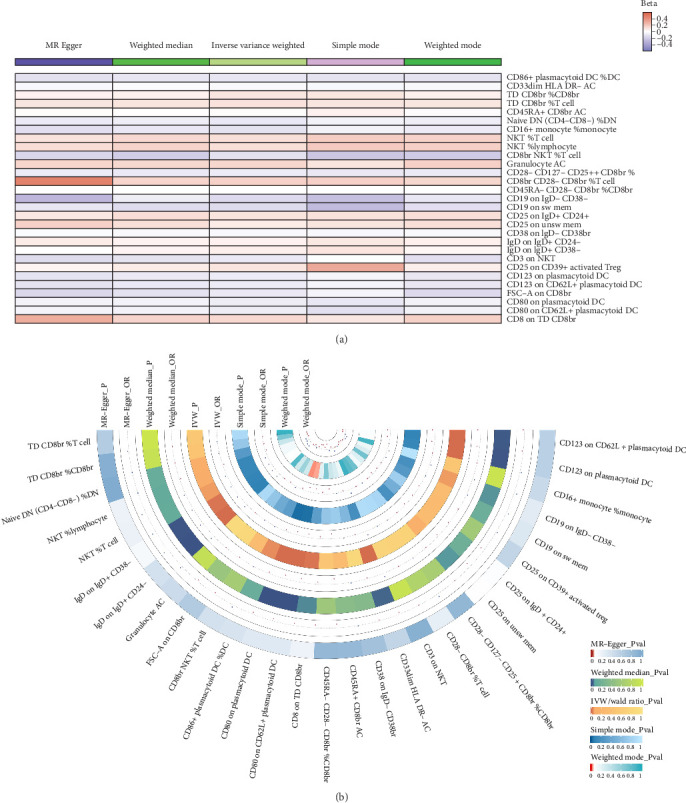
Heatmap. (A) Heatmap illustrating the relationship between the 29 identified immune cells and NAFLD. (B) Circular heatmap depicting the relationship between circulating GDF-15 levels and the 29 identified immune cells. OR, odds ratio.

A total of 29 immune cell phenotypes were shown to be linked to NAFLD based on IVW method estimations with *p* < 0.05 and consistent impact directions across all five approaches. Among these, 15 were associated with a reduced risk of NAFLD and 14 were associated with an increased risk of NAFLD (Figures 2B, 3 and 4A, Supporting Information 1: Tables S6 and S7).

#### 3.3.2. Causal Effect of Circulating GDF-15 Levels on the Identified Immune Cells

Among the 29 immune cell phenotypes previously identified, we found that GDF-15 had a causal relationship with 10 of them (Figures 2C and 4B, Supporting Information 1: Tables S8 and S9). Based on the consistency of effect directions, CD8 on TD CD8br and CD19 on IgD- CD38- were excluded, leaving a total of 8 potential mediating immune cell phenotypes (Supporting Information 1: Table S10).

#### 3.3.3. Mediation Analysis of Immune Cells

The mediation analysis's findings imply that peripheral immune cells contribute to the effects between circulating GDF-15 levels and NAFLD. Based on the selection criteria, we initially identified eight potential GDF-15—immune cell—NAFLD pathways (Supporting Information 1: Table S11). Using the delta method, we identified four immune mediators with *p*-values for the mediation effect <0.05. Specifically, GDF-15 facilitated NAFLD by downregulating the number of CD123 on plasmacytoid DCs, with a mediated effect of 11.69%. Additionally, GDF-15 exerted a detrimental effect on NAFLD by modulating the number of CD123 on CD62L+ plasmacytoid DCs, which mediated 11.71% of the total effect. Furthermore, GDF-15 also contributed to the development of NAFLD by reducing the number of CD80 on plasmacytoid DCs and CD80 on CD62L+ plasmacytoid DCs, mediating 13.98% and 13.95% of the total effect, respectively (Table 1).

### 3.4. Reverse MR Analysis

No significant reverse results were found, suggesting the stability of our mediating relationships (Supporting Information 1: Table S12–S15).

### 3.5. ScRNA-Seq Analysis

**Figure 5:**
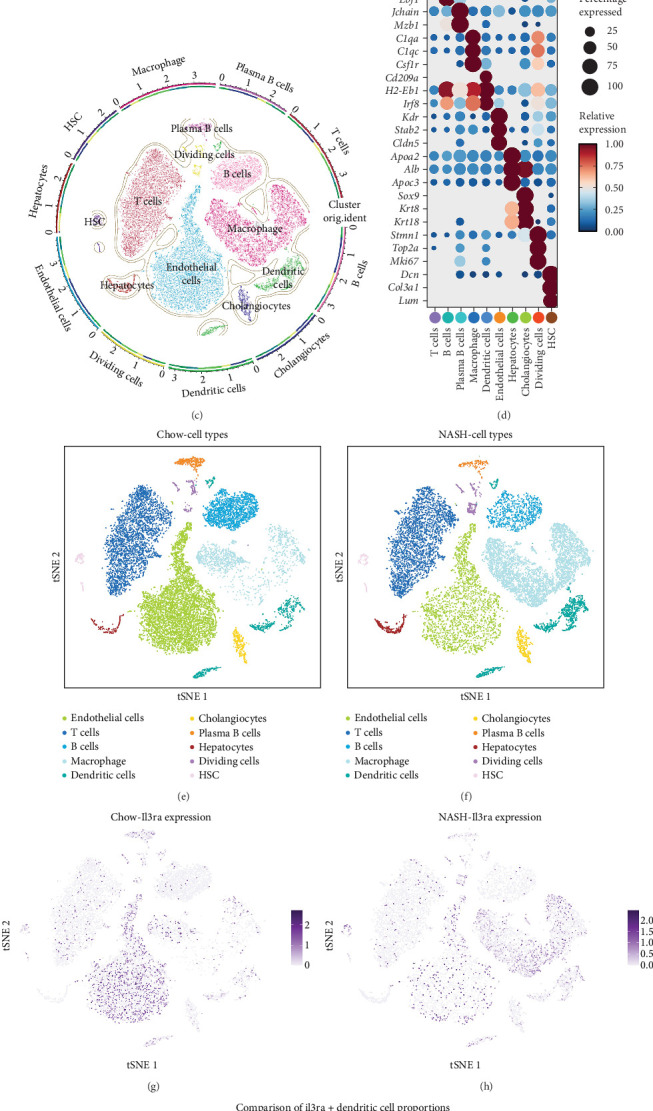
Single-cell RNA sequencing identifies Il3ra^+^ dendritic cell depletion in NASH livers. (A) t-SNE visualization of single-cell clustering. (B) Cell clusters at resolution 0.4. (C) Cell type annotation by canonical markers. (D) Cell type distribution across samples. (E–H) Il3ra expression across annotated cell types. (I) Proportion of Il3ra^+^ dendritic cells in CHOW vs. NASH groups.

High-quality cells from the six samples selected from the GSE129516 dataset were subjected to standard normalization and subsequently clustered into 18 distinct groups at a resolution of 0.4. The resulting clusters were visualized using t-distributed stochastic neighbor embedding (t-SNE) (Figure 5A,B). Cell types were annotated based on canonical marker genes, identifying nine distinct populations: T cells, B cells, plasma B cells, macrophages, DCs, endothelial cells, hepatocytes, cholangiocytes, dividing cells, and hepatic stellate cells (HSCs, Figure 5). Following annotation, we further assessed the cell type-specific expression pattern of Il3ra (Figure 5). Il3ra was broadly expressed across all cell types, with the proportion of Il3ra^+^ DCs being lower in the NASH group compared to the CHOW group (12.9% vs. 17.3%, *p* < 0.05) (Figure 5I). These findings further support the notion that GDF-15 promotes NAFLD progression by downregulating CD123 expression in plasmacytoid DCs.

## 4. Discussion

In recent years, the intricate relationship between GDF-15 and various common clinical diseases has garnered considerable attention. This study is the first to leverage a two-sample MR framework with a substantial cohort of 505,788 individuals to explore the causal association between GDF-15 and NAFLD. Our results demonstrate that elevated GDF-15 is causally linked to an increased risk of NAFLD, a finding validated by replication analyses. Furthermore, we focused on the mediating role of immune cells in the GDF-15–NAFLD axis. Using a two-step MR mediation approach, we identified four immune cell phenotypes as potential mediators of this causal relationship, offering critical insights into the prevention and therapeutic strategies for NAFLD.

Recent studies have underscored the crucial function of GDF-15 in glucose and lipid metabolism, with growing interest in its association with fatty liver disease [36, 37]. Galuppo et al. [38] reported significantly elevated plasma GDF-15 levels in obese adolescents with NAFLD compared to those without NAFLD in a cohort of 175 individuals. Koo et al. [9] found that GDF-15 levels were considerably greater in NASH patients than in control participants and NAFLD patients in a biopsy-confirmed NAFLD cohort. Furthermore, in individuals with advanced fibrosis, GDF-15 levels were even higher, and GDF-15 overexpression was found to induce phosphorylation of fibrosis-related factors in HSCs, thereby promoting liver fibrosis and accelerating NAFLD progression. Tanno et al. [39] suggested that GDF-15 suppresses hepcidin secretion in hepatocytes, triggering cell apoptosis and thereby contributing to NAFLD progression. Additionally, research by Peng Qi indicated that GDF-15 plays a profibrotic role in liver fibrosis in mice via the TGF-β/Smad2/3 pathway, proposing that inhibiting GDF-15 may serve as a viable treatment approach for alleviating fibrosis and delaying HSC activation [10]. While increasing evidence supports the profibrotic function of GDF-15 in the liver, some contradictory findings exist. One study suggested that GDF-15 overexpression promotes fatty acid β-oxidation gene expression while suppressing fibrosis-related gene expression, thereby improving NASH [40]. These discrepancies underscore the limitations of observational research, which is susceptible to reverse causation and confounding variables. Our MR analysis robustly demonstrates that elevated circulating GDF-15 levels causally promote the development of NAFLD, with no evidence of reverse causality found in bidirectional MR analyses. This further reinforces the profibrotic function of GDF-15 in liver fibrosis progression.

Despite the fact that metabolic problems are the initial cause of NAFLD, immune cells are crucial to its pathophysiology. Therefore, we further focus on the critical role of immune cells as mediators. Research has confirmed that the progression of NAFLD is closely associated with macrophages (Kupffer cells), neutrophils, DCs, NKT cells, and regulatory T cells (Tregs [41]). Research has demonstrated that lipotoxicity causes the development of NAFLD through endoplasmic reticulum (ER) stress, oxidative stress, autophagy, and lipotoxic cell death [42]. These mechanisms ultimately lead to hepatocyte death and the release of cytokines and chemokines, which draw in and stimulate immune cells to regulate the onset and severity of NAFLD [43]. Our MR analysis also confirmed that the phenotypes of 29 immune cell subsets are closely associated with the occurrence of NAFLD. Among these immune cells, the role of DCs has garnered increasing attention. DCs, as antigen-presenting cells that connect innate and adaptive immunity, play a role in limiting inflammation during the clearance of apoptotic or necrotic cells [44]. Research by Henning et al. [45] suggests that DCs may alleviate the inflammatory response in NAFLD by clearing necrotic debris and apoptotic bodies in the liver. In vivo studies have shown that the depletion of CD103, a marker of DCs, increased the severity of NASH in mice, suggesting a protective role of DCs in the development of NAFLD [46]. Furthermore, other studies have found that DCs may balance the functions of Th17 cells and Tregs in the liver, thereby influencing the progression of liver fibrosis in NAFLD patients [47]. The protective effect of DCs in NAFLD was further supported by our MR analysis, which showed that five DC phenotypes were linked to a lower incidence of NAFLD.

Previous reports have established the direct impact of GDF-15 on immune cells, and the relationship between GDF-15 and DCs has been widely studied [48]. According to one study, GDF-15 is a potent inhibitor of DC maturation, which may impair DC maturation and function by interacting with the surface receptor CD44 and suppressing its expression. This interaction can disrupt antigen uptake, processing, and presentation, leading to reduced activation of naïve T cells and impaired initiation of adaptive immune responses. Moreover, by inhibiting DC maturation, GDF-15 may promote an immunosuppressive microenvironment, potentially affecting inflammatory regulation and tolerance, and thereby influencing the progression of metabolic and inflammatory diseases such as NAFLD [49]. Zhou et al. [50] proposed that GDF-15 might inhibit the formation of surface protrusions during DC maturation, promote TGF-β1 secretion, suppress T cell activation, and reduce cytotoxic T lymphocyte activation, thereby attenuating immune responses. When combined, these results suggest that GDF-15 may increase the risk of NAFLD by modulating DC-mediated immune responses. We hypothesize that GDF-15 exerts its effect by inhibiting DC maturation and reducing the expression of surface markers, thereby altering the liver's immune environment and promoting inflammation, which in turn increases the risk of NAFLD. Our results provide valuable insights into the potential mediating role of DCs in the pathway from GDF-15 to NAFLD, although the precise mechanisms remain to be further elucidated, and the modest mediation proportion (11%–14%) warrants cautious interpretation.

In this study, we propose for the first time that DCs mediate the causal relationship between GDF-15 and NAFLD. This novel finding provides new perspectives on NAFLD management and carries several important clinical implications. First, by identifying DCs as key mediators of the GDF-15–NAFLD axis, we provide a compelling rationale for targeting this immunometabolic pathway for therapeutic intervention. Agents that modulate GDF-15 signaling or restore the functional integrity of DCs could represent a novel class of therapeutics for NAFLD. Second, our findings suggest that circulating GDF-15 levels and specific DC subsets (e.g., CD123^+^ pDCs) may serve as valuable biomarkers for early identification of individuals at high risk for NAFLD progression, enabling timely preventive strategies. Finally, as drugs targeting the GDF-15 pathway are already under development for metabolic diseases, our results directly inform and potentially accelerate their repurposing for the treatment of NAFLD. However, several limitations should be addressed. First, our analysis relied exclusively on European datasets, which may limit the generalizability of our results to other ethnic populations. Second, the study did not encompass all potential immune cell phenotypes, implying that the function of other unstudied immune cell phenotypes in the GDF-15–NAFLD axis cannot be excluded. Third, multiple testing correction was not applied in the analyses of immune cell phenotypes because this study was designed as an exploratory analysis, aiming to identify as many potential associations as possible; therefore, the risk of false-positive findings may be increased. Fourth, the identified mediator immune cell phenotypes were not further validated using independent GWAS datasets, which limits the replication and robustness of these findings. Additionally, while our MR analysis provides preliminary evidence for the causal link between GDF-15 and NAFLD, with DCs serving as mediators, these conclusions remain unsupported by animal or clinical evidence, which may be a focal point for future investigations. Finally, although liver fibrosis is a critical aspect of NAFLD progression, we were unable to test the causal relationship between GDF-15 and fibrosis outcomes due to the lack of publicly available GWAS data on NAFLD fibrosis stages, which represents an important avenue for future research.

## 5. Conclusion

In summary, our study found that elevated levels of GDF-15 are associated with an increased risk of NAFLD, with DCs playing a crucial mediatory role in this context. These findings lay the groundwork for future research and offer novel targets and pathways for therapeutic interventions in NAFLD.

## Figures and Tables

**Table 1:** The mediation effect of circulating GDF-15 levels on NAFLD via immune cells.

Exposure	Mediator	Outcome	Total effect	Mediation effect	*p*-Value	Mediated proportion (%)
			β (95% CI)	β1*⁣*^*∗*^β2 (95% CI)		
GDF-15	CD123 on plasmacytoid DC	NAFLD	0.145 (0.026–0.263)	0.017 (0.001–0.033)	0.040	11.690%
GDF-15	CD123 on CD62L + plasmacytoid DC	NAFLD	0.145 (0.026–0.263)	0.017 (0.001–0.033)	0.040	11.705%
GDF-15	CD80 on plasmacytoid DC	NAFLD	0.145 (0.026–0.263)	0.020 (0.002–0.038)	0.028	13.977%
GDF-15	CD80 on CD62L + plasmacytoid DC	NAFLD	0.145 (0.026–0.263)	0.020 (0.002–0.038)	0.029	13.955%

Abbreviations: NAFLD, nonalcoholic fatty liver disease; GDF-15, growth differentiation factor 15.

## Data Availability

The entirety of the data examined in the present study can be accessed through the GWAS Catalog as well as the IEU OpenGWAS database.

## Nomenclature

cDC: Conventional dendritic cells
CVD: Cardiovascular disease
GDF-15: Growth differentiation factor 15
GWAS: Genome-wide association study
HCC: Hepatocellular carcinoma
IVs: Instrumental variables
IVW: Inverse variance weighted
MR: Mendelian randomization
NAFLD: Nonalcoholic fatty liver disease
NASH: Nonalcoholic steatohepatitis
OR: Odds ratio
scRNA-seq: Single-cell RNA sequencing
TBNK: T cells, B cells, and natural killer cell
Treg: Regulatory T cell.

## Supporting Information

Supporting Information 1 Table S1: Data sources of GWAS summary obtained for MR analyses. Table S2: Characterization of immunophenotypes. Table S3: Summary of five SNPs significantly associated with the circulating GDF-15 levels. Table S4: Summary of 11,836 SNPs significantly associated with the 731 immune cells. Table S5: Mendelian randomization analysis of the causal effect of GDF-15 on NAFLD using supplementary robust methods (cML-MA, MR-ConMix, and MR-RAPS). Table S6: Significant causal associations between immune cells on NAFLD. Table S7: Sensitivity analysis between immune cells on NAFLD. Table S8: Significant causal associations between the circulating GDF-15 levels and the identified immune cells. Table S9: Sensitivity analysis between the circulating GDF-15 levels and the identified immune cells. Table S10: Screening of immune cells as potential mediators in the causal pathways from GDF-15 to NAFLD. Table S11: Mediation analysis. Table S12: Summary of 28 SNPs significantly associated with the NAFLD. Table S13: MR results of NAFLD on the circulating GDF-15 levels. Table S14: MR results of NAFLD on the identified immune cells. Table S15: MR results of the identified immune cells on the circulating GDF-15 levels.

Supporting Information 2 Figure S1: Scatter plots of estimates for associations between circulating GDF-15 levels on NAFLD. Causal influence is represented by the slope value, which is equal to the *b*-value computed using the five methods. A positive slope indicates a risk factor for exposure. (A) Primary analysis. (B) Replication analysis. Figure S2: Leave-one-out plots for associations between circulating GDF-15 levels on NAFLD. (A) Primary analysis. (B) Replication analysis.
