# Supplementary material for: Role of Dendritic Cells in Mediating the Effect of Growth Differentiation Factor 15 on Nonalcoholic Fatty Liver Disease: Insights From Causal Inference and Single-Cell Profiling
Source: Mediators Inflamm. 2025 Nov 24;2025:1153091. doi: 10.1155/mi/1153091 (PMC12668855; doi:10.1155/mi/1153091)

Supplementary figure

Figure S1 Scatter plots of estimates for associations between circulating GDF-15 levels on NAFLD. Causal influence is represented by the slope value, which is equal to the b-value computed using the five methods. A positive slope indicates a risk factor for exposure. (A) Primary analysis. (B) Replication analysis.

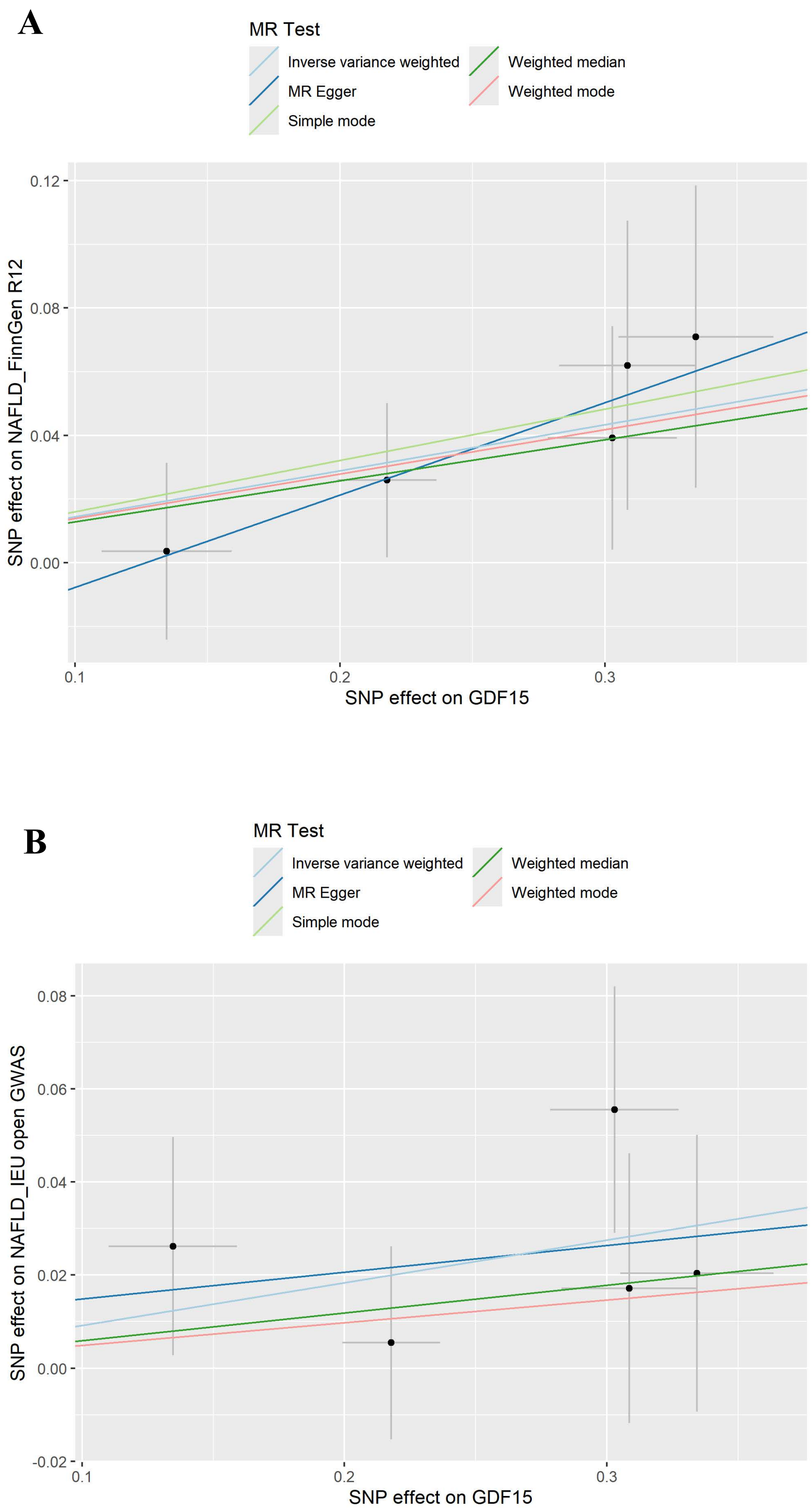

Figure S2 Leave-one-out plots for associations between circulating GDF-15 levels on NAFLD. (A) Primary analysis. (B) Replication analysis.

A

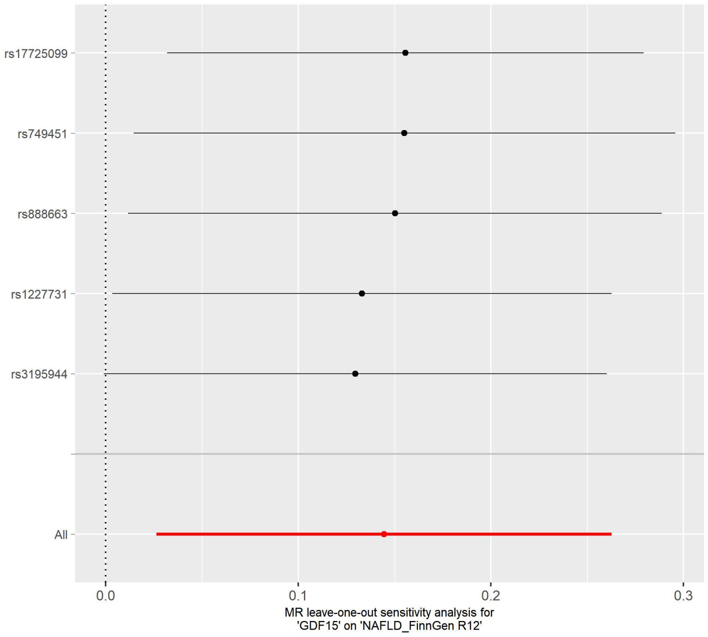

B

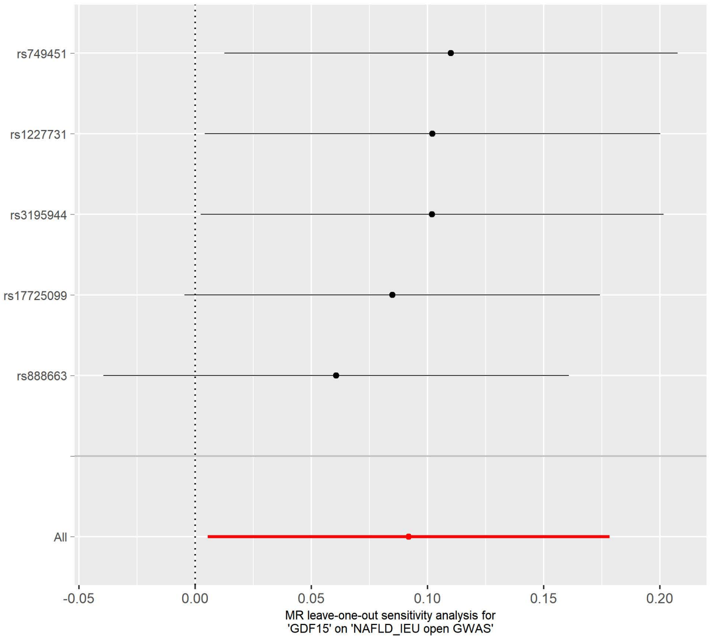

Supplement: Supporting Information 2 — Figure S1: Scatter plots of estimates for associations between circulating GDF-15 levels on NAFLD. Causal influence is represented by the slope value, which is equal to the b-value computed using the five methods. A positive slope indicates a risk factor for exposure. (A) Primary analysis. (B) Replication analysis. Figure S2: Leave-one-out plots for associations between circulating GDF-15 levels on NAFLD. (A) Primary analysis. (B) Replication analysis. [file 1153091.f2.pdf]
